# Ethnicity and COVID-19 infection: are the pieces of the puzzle falling into place?

**DOI:** 10.1186/s12916-020-01669-9

**Published:** 2020-07-01

**Authors:** Rachel H. Mulholland, Ian P. Sinha

**Affiliations:** 1 Usher Institute, The University of Edinburgh, Edinburgh, UK; 2 Alder Hey Children’s Hospital, Liverpool, UK; 3 University of Liverpool, Liverpool, UK

**Keywords:** COVID-19, Ethnic inequalities, UK Biobank, Public health, Epidemiology

## Background

The COVID-19 pandemic is exposing problems within our societies, and as always, vulnerable groups are hit hardest. One key story around COVID-19, particularly in the UK and USA, has been inequality in COVID-19 outcomes between people from different ethnic backgrounds. Reports of such differences initially emerged from certain states in the USA, where rates of hospitalisation and death from COVID-19 were higher in Black people than would be expected from the ethnic composition of the population [1]. Similar findings are shown in the UK, where Black and South Asian people have disproportionately high rates in intensive care [2], mortality amongst healthcare workers [3] and the general population [4], and the development of paediatric inflammatory multisystem syndrome (a paediatric hyperinflammatory state related to COVID-19) [5]. The question is, why?

## Linking data between UK Biobank and Public Health England

The linked paper by Niedzwiedz et al. [6] adds much-needed new information. This study links participants in the UK Biobank to COVID-19 testing data from Public Health England (PHE). The study recruited 40–70-year-olds and recorded their baseline characteristics between 2006 and 2010 [7]. Associations between the baseline data, in particular, self-reported ethnicity and socioeconomic status (deprivation and highest education), were explored and compared in COVID-19 testing, testing positive and testing positive in a hospital setting. Results were adjusted for potential confounding and mediating factors that reflected the health and social status of the participants.

Of 392,116 participants in the cohort, 2658 were tested for COVID-19, of whom 948 tested positive. Compared to the White British population, positive tests were more likely in Black (relative risk (RR) 3.35 (95% CI 2.48–4.53)), South Asian (RR 2.42 (95% CI 1.75–3.36) and White Irish (RR 1.42 (95% CI 1.00–2.03)) people. When breaking down into more detailed categories, Black Caribbean (RR 3.51 (95% CI 2.39–5.15) and Pakistani (RR 3.24 (95% CI 1.73–6.07)) ethnic groups had respectively the highest risk ratio in the Black and South Asian ethnic backgrounds. The risk of a positive test was also linked to socioeconomic status and education, where those living in the most disadvantaged quartile (RR 2.19 (95% CI 1.80–2.66)) and those with lower levels of education (RR 2.00 (95% CI 1.66–2.42)) having the highest risk of confirmed COVID-19 infection. Adjusting for potential risk factors, including country of birth, whether they are a healthcare worker, socioeconomic status and pre-existing health, did not fully explain these ethnic differences. Socioeconomic differences, however, appeared to make the most substantial contribution to these ethnic variations.

Collaborations such as these are needed to address public health emergencies, and all parties (the authors, Biobank UK and PHE) should be commended for enabling and conducting a linked data study of this scale so quickly. Insights are extremely useful and further evidence of what has been previously seen in ethnic groups and socioeconomic backgrounds in relation to COVID-19.

Nonetheless, there are potential limitations to cohort studies using UK Biobank. Firstly, the cohort consists of volunteers meaning results may not reflect the whole UK with complete accuracy. In particular, this study only uses participants from English assessment centres who were aged 40–70 years when recruited and therefore cannot be generalised nationally. Secondly, the baseline characteristics of participants were recorded over 10 years ago and may have changed since they were enrolled. In addition, the models only adjust for risk factors and do not explore potential interactions between the exposures and risk factors. Investigating these may add extra value to insights to evaluate whether ethnic differences occur differently in the potential confounding factors. Alongside this analysis, further data from people in hospitals and care homes would help identify where along the patient pathways the problems lie.

## Where next?

In order for population-level interventions and policies to be impactful, we urgently need to develop a mechanistic understanding of the increased risk to different communities. A holistic approach should be adopted, alongside more detailed and up-to-date information around social issues that differ between ethnic groups, such as environmental conditions (including overcrowding, intergenerational living, standards of accommodation and exposure to pollution), income, type of employment and working conditions. Linking data with health records would enable a more accurate exploration of the role of relevant comorbidities—not just in terms of prevalence and disease control but also the quality of care. Public health messaging to different ethnic groups requires examination, to ensure it is clear and culturally appropriate at the local and governmental levels. There is interest in genetic and biological reasons that explain these ethnic inequalities, but to date, this is only speculative. Theories around the expression of human leukocyte antigen haplotypes, angiotensin-converting enzyme receptors and vitamin D levels in dark-skinned people have not been validated. The role of these factors in COVID-19 disease is poorly understood, and no group has yet linked them with ethnicity and COVID-19 outcome in a meaningful way to explain the stark inequalities [8–10].

## Conclusion

Research on ethnic inequalities from COVID-19 is still lacking, and most countries with high levels of infection have not yet released data around this issue. Further qualitative and quantitative research, grounded in public health and social sciences, is urgently needed. Resources like the UK Biobank will enable both epidemiological and biological research, and the linked study is an example of how useful studies can be done rapidly. The ethnic inequalities in COVID-19 are of grave importance. The response needs to be collaborative, meaningful, transparent, sensitive, holistic and swift.

## Data Availability

Not applicable

## Abbreviations

COVID-19: Coronavirus disease 2019
PHE: Public Health England
RR: Relative risk
